# Divergent jejunal *cis*- and *trans*-eQTLs and their microbiome associations following a diet lacking mineral phosphorus supplements in laying hens

**DOI:** 10.1186/s12864-026-13096-w

**Published:** 2026-06-20

**Authors:** Yosef A. Abitew, Henry Reyer, Michael Oster, Valentin P. Haas, Markus Schmid, Vera Sommerfeld, Yi-Hsuan Chien, Amélia Camarinha-Silva, Jörn Bennewitz, Markus Rodehutscord, Klaus Wimmers, Siriluck Ponsuksili

**Affiliations:** 1 Research Institute for Farm Animal Biology (FBN), Wilhelm-Stahl-Allee 2, Dummerstorf, 18196 Germany; 2 Institute of Animal Science, University of Hohenheim, Stuttgart, Germany; 3 Faculty of Agricultural, Civil and Environmental Engineering, University of Rostock, Rostock, Germany

**Keywords:** Mineral phosphorus, eQTL, Intestinal tract, Laying hen, Microbiota

## Abstract

**Background:**

Phosphorus (P) utilization is a complex trait influenced by numerous genetic variants. The jejunum is the primary site of P absorption in poultry. Therefore, identifying the genetics that regulate transcription in jejunum may help uncover key regulators of P homeostasis. We performed a genome-wide association study using the expression of jejunal mucosa transcripts (88 miRNAs, 65 mRNAs). These transcripts were selected from our previous studies due to their association with P utilization and related pathways. In total, the trial comprised 400 laying hens from two high-yielding strains, Lohmann Brown (LB) and Lohmann Selected Leghorn (LSL), fed a diet lacking mineral P supplements and exogenous microbial phytase to stimulate adaptive mechanisms.

**Results:**

In total, 114 miR-eQTLs (microRNA expression quantitative trait loci) were detected at a false discovery rate (FDR) of less than 5%, including 56 miR-eQTLs in the LB strain and 58 miR-eQTLs in the LSL strain. Lohmann Brown contained 23 *cis* and 35 *trans* loci, with the most significant *cis*-eQTL targeting miR-146b. In the LSL strain, a *cis*-eQTL cluster for miR-203a was present on chromosome 5. Similarly, 123 mRNA-eQTLs (94 in LB and 29 in LSL) were identified at the 5% FDR threshold. The genetic regulation of key genes involved in mineral binding and mineral transport, including *CALB1* and *SLC34A2*, in LB hens was predominantly driven by strong *cis*-eQTL. In contrast, gene expression in LSL hens was largely modulated by *trans*-eQTLs, with *CALM1* being the only gene under significant *cis*-regulation. Furthermore, correlation analysis with the gut microbiome revealed that the expression of *cis*-regulated *CALB1* is significantly positively associated with the abundance of *Lactobacillus* species.

**Conclusions:**

Our findings reveal that LB and LSL hens exhibit distinct genetic architectures contributing to maintain mineral homeostasis. Genetic differences between the two strains influence the transcriptional response of key mineral transporter genes and miRNAs under a low-P diet. These divergent host genetic strategies are also associated with distinct gut microbiota profiles, highlighting interactions between host genetics, gene expression, and the microbiome in P utilization.

**Supplementary Information:**

The online version contains supplementary material available at 10.1186/s12864-026-13096-w.

## Background

Phosphorus (P) is a crucial nutrient for laying hens and necessary for bone development, eggshell formation, and overall health. However, the primary source, i.e., mineral phosphate rock, is a non-renewable resource [1]. A central paradox in laying hen nutrition is the handling of P [2]. Although P is essential for the health and productivity of laying hens, the P in their plant-based diet is present as largely indigestible phytate [3]. As laying hens lack sufficient endogenous phytase or phosphatases [4], their feed is usually supplemented with mineral P or phytase of microbial origin or both [5]. In commercial laying hen diets, mineral P might be present at levels that exceed the actual physiological demand of the birds [6].

Improving the genetic potential of hens to utilize dietary P more efficiently might be a sustainable alternative [7], as it is well documented that the efficiency with which different laying hen strains utilize P varies. Specifically, studies comparing Lohmann Brown (LB) and Lohmann Selected Leghorn (LSL) hens have revealed differences in P retention, endogenous phosphatase activity, intestinal phosphate transporter expression, and the circulating levels of hormones that regulate P and Ca homeostasis, such as calcitriol and estradiol [5, 8–10]. Recent studies have confirmed this finding, revealing distinct strain-specific physiological responses to low P supplies, ranging from differences in gastrointestinal phytate degradation [8] to unique gene expression patterns in the kidney and jejunum [10, 11] and overall P utilization efficiency [12]. These physiological, molecular, and quantitative-genetic differences strongly suggest differences in the underlying genomic mechanisms between the two strains.

The gut environment, microbiome composition, and host-microbiota interactions all play significant roles in this process. Microbial phosphatases contribute to the release of P from inositol phosphates (InsP) in the gut of poultry [13]. MicroRNAs (miRNAs) are a class of small noncoding RNA molecules that function as key posttranscriptional regulators of gene expression [14]. In the gastrointestinal tract, the host’s genetics can influence the expression of both genes and miRNAs. miRNAs are important mediators in the two-way interaction between the host and its gut microbiota. For instance, microbiome stimulation in the early life of poultry can alter host miRNA expression in immune tissues [15]. Meanwhile, specific host-derived miRNAs have been shown to shape gut bacteria, directly regulating microbial gene expression and metabolic function [16]. Expression quantitative trait locus (eQTL) mapping, also known as genetical genomics, detects genetic variants that influence gene expression traits [17]. Large-scale initiatives, such as the Chicken Gene-Tissue Expression (ChickenGTx) consortium, have mapped the genetic basis of mRNA expression across various chicken tissues [18].

A previous study in an F2 Japanese quail population identified mainly *cis*-acting miRNA eQTLs and mRNA eQTLs and integrated these loci with the gut microbiome and phenotypic data, particularly regarding P efficiency [19]. In addition, microbiota diversity has been reported to impact P efficiency in poultry models in a causal manner [20] and directional relationships between certain microbial genera and host efficiency traits related to P utilization were detected [21]. However, the regulatory roles of both mRNA and miRNA eQTLs in the gut of laying hens under a low-P diet remain largely unknown, as does their interplay with the gut microbiome.

In this study, we performed comprehensive eQTL analyses using expression profiles for a targeted group of miRNAs and mRNAs from the jejunal mucosa. These transcripts were selected for quantification by qPCR based on our previous findings and their established role in key biological metabolic pathways, including InsP metabolism and the phosphatidylinositol signaling system [22–24]. By integrating these data with gut microbiome profiles of the same animals, we aimed to better characterize causal transcripts linked to P metabolism, as well as genetically regulated transcripts associated with the intestinal microbial abundance in two high yielding laying hen strains.

## Methods

This study was part of the interdisciplinary Research Unit P-Fowl: Inositol phosphates and *myo*-inositol in the domestic fowl: Exploring the interface of genetics, physiology, microbiome, and nutrition (https://p-fowl.uni-hohenheim.de). The animal trial was approved by the Regierungspräsidium Tübingen (regional administrative authority in the state of Baden-Württemberg), Germany (Project no. HOH67-21TE) in accordance with the German Animal Welfare Legislation.

### Experimental design and sample collection

The experimental cohorts were established using two commercial laying hen strains, LB and LSL, which represent two distinct genetic backgrounds. For each strain, 10 non-related roosters were used for egg production. A total of 648 hens were raised and 400 hens (200 per strain) were used for the experiment. Initially, the birds were raised in floor pens on deep litter bedding including a comprehensive vaccination program. Once high laying performance was attained the hens were assigned to four subsequent cohorts to receive experimental diets for a period of 3 weeks. Experimental diets were formulated that comprised adequate levels of all nutrients according to the recommendations of the Gesellschaft für Ernährungsphysiologie (GfE) [25], except P, lacking mineral P supplements and exogenous phytase. Feed and water were provided for *ad libitum* consumption. At the age of 27, 31, 35, and 39 weeks, 100 hens (50 LB; 50 LSL) were moved to individual metabolic units. Birds were slaughtered at 30, 34, 38, and 42 weeks of age (cohorts 1–4, respectively). All birds were clinically healthy prior to and at the time of sampling. Prior to slaughter, birds were fasted for 1 h and then given 1 h of *ad libitum* access to feed to standardize gut fill. The hens were individually stunned with a gas mixture consisting of 35% carbon dioxide, 35% nitrogen dioxide, and 30% oxygen. The hens were then killed by bleeding. Approximately 5 cm of the proximal jejunum were sampled. The intestinal sections were flushed with ice-cold 0.9% NaCl solution. For the eQTL analysis, the jejunal mucosa was sampled via microscopic slides, shock-frozen on dry ice, and subsequently stored at -80° C until RNA extraction. For the microbiota composition analysis, digesta was collected from the duodenum of the same animals.

### miRNA and mRNA extraction, sequencing, and quantitative PCR

The objective of this study was to identify genetic variants associated with jejunal miRNA and mRNA expression under a low-phosphorus diet. Candidate transcripts for eQTL mapping were selected based on our previous studies using a smaller subset of animals from the same genetic lines exposed to different dietary phosphorus strategies [22–24]. These earlier analyses enabled us to identify mRNAs and miRNAs that were most responsive to dietary phosphorus supply and therefore most relevant for targeted eQTL analysis. Selection was based on two main criteria: strong differential expression under low-phosphorus conditions and/or involvement in pathways central to phosphorus biology, including inositol phosphate metabolism, the phosphatidylinositol signaling system, oxidative phosphorylation, lipid metabolism, and immune signaling. These candidate transcripts were then quantified by quantitative polymerase chain reaction (qPCR) across all 400 animals to generate expression phenotypes for eQTL mapping. The Fluidigm BioMark HD system was chosen because it enables high-throughput, simultaneous quantification of up to 96 target transcripts, providing precise and reproducible expression measurements for quantitative genetic analysis at substantially lower cost than transcriptome-wide RNA-seq in all individuals. In total, 88 miRNAs and 65 mRNAs were quantified using 96.96 Dynamic Arrays (Fluidigm Corporation, San Francisco, CA). The specific target amplification (STA) procedure was performed according to the manufacturer’s guidelines. Preamplification sample mixtures were prepared via PreAmp Master Mix (Fluidigm PN 1005581) containing 1.25 µl of cDNA, 1 µl of PreAmp Master Mix, and 0.5 µl of pooled delta gene assay mixture (500 nM) containing DNA suspension buffer and primer mixtures in a 5 µl total volume. The preamplification mixture was then incubated at 95 °C for 2 min, followed by 10 cycles at 95 °C for 15 s and 60 °C for 4 min. The preamplification reaction was then subjected to exonuclease I treatment, followed by a 10-fold dilution of STA with DNA suspension buffer (TEKnova, PN T0221). The quantitative measurement runs were conducted via 96.96 dynamic arrays (Fluidigm Corporation) according to the manufacturer’s guidelines. In summary, 2.5 µl of 2 × SsoFast Evagreen Supermix with Low ROX, 0.25 µl of 20 × sample-loading reagent, and 2.25 µl of treated samples were prepared. Concurrently, an assay mixture comprising 2.25 µL of DNA suspension buffer, 0.25 µL of 100 µM forward and reverse primers, and 2.5 µL of 2× assay-loading reagent was prepared for each primer pair.

The dynamic arrays were initially primed with control line fluid and subsequently loaded with the sample and assay mixtures via the designated inlets via an IFC controller. Afterward, the array chips were placed in the BioMark instrument for PCR, utilizing the following thermal cycling program. The initial step was to subject the sample to a temperature of 95 °C for 10 min. This was followed by 30 cycles at 95 °C for 15 s and 60 °C for one minute. Quantitative PCR was performed in 4 batches covering a balanced number of samples from strains and cohort age. The subsequent analysis of the data was conducted via the real-time PCR analysis software in the BioMark HD instrument (Fluidigm Corporation).

### Expression data preprocessing and normalization

Normalization of the miRNA and mRNA expression levels were performed by fitting a linear model via a standard curve approach. Specifically, standard curves were generated by preparing a series of known copy numbers of synthetic miRNA and mRNA standards, which were analyzed alongside our experimental samples by plotting the cycle threshold (Ct) values against the logarithm of the known concentrations. This process established a linear regression model for each target, allowing the accurate quantification of expression levels in the experimental samples. To account for technical variability, such as differences in RNA input or reverse transcription efficiency, the data was further normalized using the geometric means of selected reference genes (cel-miR-39-3p, Quail-18 S and *SNORD21* for miRNA; and *ACTB*, *GAPDH*, *RPL13*, and *TBP* for mRNA). The efficiency and linearity of the standard curves were validated, ensuring that only those with PCR efficiencies between 90% and 110% and R² values above 0.95 were used for subsequent quantification. Ten 96 × 96-array chips were processed. To minimize confounding between chip and cohort, each chip included samples from all cohorts, and chip identity was modeled as a batch-effect in the subsequent analysis. Finally, normalized expression values were log_10_-transformed to stabilize variance across samples. The primer sequences used in this study are listed in Table S1 of Additional File 1.

### Genotyping and variant quality control

A total of 400 experimental samples were designated for genotyping via the 60 K chicken Infinium iSelect chip. Following data quality control, one LB sample was excluded, with a final dataset of *n* = 399 genotyped birds (LB = 199, LSL = 200), which resulted in a genotyped array of 57,636 single-nucleotide polymorphisms (SNPs). At the sample level, individuals with a genotype missing rate greater than 5% were excluded. At the SNP level, quality control was performed sequentially. Variants were filtered by call rate (≥ 0.95; all SNPs passed), cluster separation (≥ 0.4; 2,023 SNPs removed), call frequency (≥ 0.95; 594 SNPs removed), and heterozygosity excess (− 0.3 to 0.3; 17,408 SNPs removed), resulting in 37,611 SNPs. Additional SNP filtering was then applied separately to the LSL, LB, and pooled datasets. Within each analysis cohort, variants were excluded if they deviated from Hardy–Weinberg equilibrium (HWE < 1 × 10⁻⁶) or had a minor allele frequency (MAF) below 0.05, as determined using PLINK v1.9.0 [26]. This process resulted in 14,709 SNPs for the LB analysis, 10,240 SNPs for the LSL analysis and 37,611 SNPs for the pooled analysis. The genetic map was updated to Gallus Gallus 6 (GRCg6a) assembly via the biomaRt R package (version 2.64.0) [27]. Afterward, the genomic positions of the miRNAs and mRNAs were retrieved from Ensembl (https://apr2022.archive.ensembl.org/Gallus_gallus/) [28], which was once more based on the GRCg6a assembly and utilizing the same biomaRt tool.

### Analysis of microbiota composition

Different intestinal compartments were used because of their biological relevance: jejunal mucosa was selected for host transcriptome analysis as the jejunum is a key site of nutrient and phosphorus absorption, whereas ileal digesta was used for microbiome profiling because it better represents the luminal microbial community. Both datasets were obtained from the same animals, with the microbiome data originating from a parallel study based on the same experimental design. DNA from ileal digesta samples was extracted using the FastDNA™ SPIN Kit for Soil (MP Biomedicals, Santa Ana, CA, USA). Shotgun metagenomic sequencing was performed on the Illumina NovaSeq 6000 platform using paired-end 150-bp reads. Raw reads were quality-filtered and adapter-trimmed using Trim Galore with default settings, including a Phred quality score cutoff of 20 and a minimum read length of 20 bp [29]. Host-derived reads were removed by aligning sequences to the chicken reference genome GRCg6a (GenBank accession GCF_000002315.6) and maize reference sequences using Bowtie2 [30]. After removal of host- and feed-derived sequences, an average of approximately 35 million high-quality reads per sample were retained for downstream analysis. Taxonomic classification of filtered reads was performed using Kraken 2 against the NCBI reference database within the QIIME 2 platform [31, 32]. Taxa at the species level were filtered to retain only those with more than one observation in at least half of the samples. The normalized microbial abundances were subsequently transformed via a centered log-ratio (CLR) transformation. These CLR-transformed values were then used for Spearman’s rank correlation with miRNA and mRNA expression data.

### Statistical analysis

Due to management-related constraints, the laying hens were sampled in separate age cohorts. Subsequent analysis showed that cohort age significantly affected expression levels (Wald test in the linear mixed model, *p* < 0.05); therefore, cohort age was included as a fixed-effect covariate in the model. For each of the 88 miRNA and 65 mRNA expression phenotypes (indexed by *i*; *n* = 399 samples) and for each genetic variant (indexed by *j*), the following univariate linear mixed model (LMM) was fitted using Genome-wide Efficient Mixed Model Association (GEMMA) [33]:

$$\mathrm{y}_{i}\;=\;W\alpha_{i}\;+\;\mathrm{x}_{j}\;\beta_{ij}\;+\;u_{i}\;+\;\varepsilon_{i}$$

where **y***i* is the *n*×1 vector for phenotype *i*, **W** is the n×c covariate matrix (batch, cohort age, strain, and the geometric mean of housekeeping-gene expression), α_i_ are fixed-effect coefficients for the covariates. These covariates were included a priori in all models to correct for known biological and technical confounders. **x***ⱼ* is the *n*×1 genotype vector for variant *j* coded additively. β*iⱼ* is the regression coefficient representing the additive genetic effect of SNP *j* on the expression of transcript *i*. Variance components for genetic ($\:{{\sigma\:}^{2}}_{\mathrm{g},i}$) and environmental ($\:{{\sigma\:}^{2}}_{e,i}$) effects were estimated by REML in GEMMA v0.98.1 using default settings [33]. Random effects were the polygenic effects **u**_i_ ~ 𝒩 (0, $\:{{\sigma\:}^{2}}_{\mathrm{g},i}$**K**) and the residuals **ε**_i_ ~ 𝒩 (0, $\:{{\sigma\:}^{2}}_{e,i}\:\mathbf{I}_\mathrm{n}$). **K** being the genomic relationship matrix estimated from centered genotypes in GEMMA, using default settings [34]). eQTL analysis was performed separately for the LSL cohort, LB cohort, and on a pooled dataset containing both strains. In strain-specific analyses, we stratified by strain, excluding the strain indicator from **W**, and estimating **K** using only samples from the corresponding strain. Association *P* values for β*iⱼ* were obtained with GEMMA’s Wald test. A 1 Mb window from the transcription start site of the specific expression phenotypes was defined for *cis*-regulatory eQTLs, and those outside this range and variants on other chromosomes (Chr) were assigned as *trans*-regulatory eQTLs. Multiple testing was controlled separately for the miRNA and mRNA analyses using Benjamini-Hochberg FDR; associations with FDR < 0.05 were called significant [35].  

## Results

### eQTLs for miRNAs (miR-eQTL)

Table 1 summarizes the number of significant *cis*- and *trans*-miR-QTLs identified in each of the two strains separately and the combined analysis of both strains. The comprehensive results of the miR-eQTL analysis are presented in Table S2 [see Additional file 1 - Table S2].

**Table 1:** Distribution of significant miR-QTLs by strain and regulatory type

Strain^a^	Total	cis	Unique cis-miRNAs^b^	trans	Unique trans-miRNAs
LB	56	15	*miR-146b*, *miR-203a*, miR-219b, miR-34a, miR-7460	41	miR-10a, miR-130a, miR-133b, mir-140, miR-199, miR-210a, miR-2188, miR-34a, miR-455, miR-726, miR-7460, miR-10c, miR-126
LSL	58	23	*miR-146b*, *miR-203a*	35	miR-146b, miR-1788, miR-30a
Both	104	21	miR-7460, miR-146b	83	miR-726, miR-203a, miR-34a, miR-146b, miR-7460, miR-191, miR-147, miR-30a

The columns indicate the hen strain, the total number of significant associations, the number of *cis*- and *trans*-acting miR-QTLs, and the corresponding distinct miRNAs targeted by these associations. The genome-wide significance threshold was set to false discovery rate (FDR) < 0.05

^a^*LB* Lohmann Brown, *LSL* Lohmann Selected Leghorn hen strains, and the combined strain analyses (Both)

^b^For the miRNAs shown in italics, associated eQTLs were identified in both strains

**Fig. 1:**
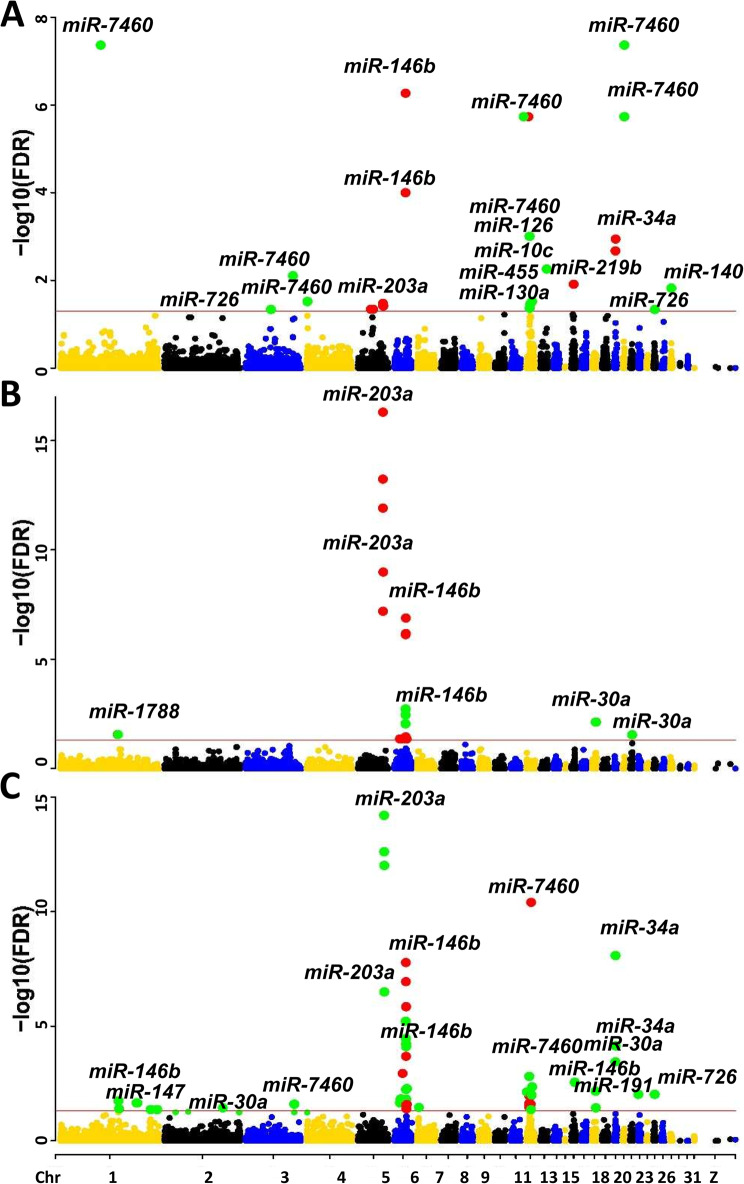
Manhattan plot of miRNA quantitative trait loci (miR-eQTLs) for 88 highly expressed jejunal transcripts laying hens. Panels display results for the (**A**) LB strain (*n* = 199), (**B**) LSL strain (*n* = 200), and (**C**) a combined analysis for both strains with the strain included as a covariate. Red points indicate significant *cis*-eQTLs (variants within 1 Mb of the target gene), whereas green points denote significant *trans*-eQTLs. The horizontal red line represents the genome-wide significance threshold (false discovery rate (FDR) < 0.05), whereas the x-axis represents the chromosomal position according to the GRCg6a assembly

In total, 88 different miRNA expression profiles were used as response variables in a genome-wide association framework implemented in GEMMA (LMM), which tested 37,611 SNPs against each miRNA phenotype. A total of 56 significant associations between miRNAs and SNPs were identified in the LB strain, and 58 in the LSL strain. Figure 1 displays the genomic locations of peak eQTL SNPs (local and distal) alongside their associated miRNA transcripts (Fig. 1A (LB), B (LSL), C (both strains)).

The LB strain presented 56 miR-eQTLs, which together regulated the expression of 20 different miRNAs. This total comprised 17 *cis*-miR-eQTLs, affecting five of these miRNAs, and 39 *trans*-miR-QTLs, affecting 12 miRNAs. The most significant association with a SNP marker in *cis* was observed for miR-146b at SNP rs317732566 (Chr 6: 24,156,386 bp; p_wald = 3.67 × 10^− 11^, β = +0.119, FDR = 5.40 × 10^− 7^, Fig. 1A). We identified 39 significant *trans*-acting SNPs that influence 12 distinct miRNAs [see Additional file 1 - Table S2]. Notably, *trans* hotspots were identified at loci on Chr 13, where the peak SNP rs316417999 at Chr 1:81831988, affecting miR-7460-3p, was located.

**Fig. 2:**
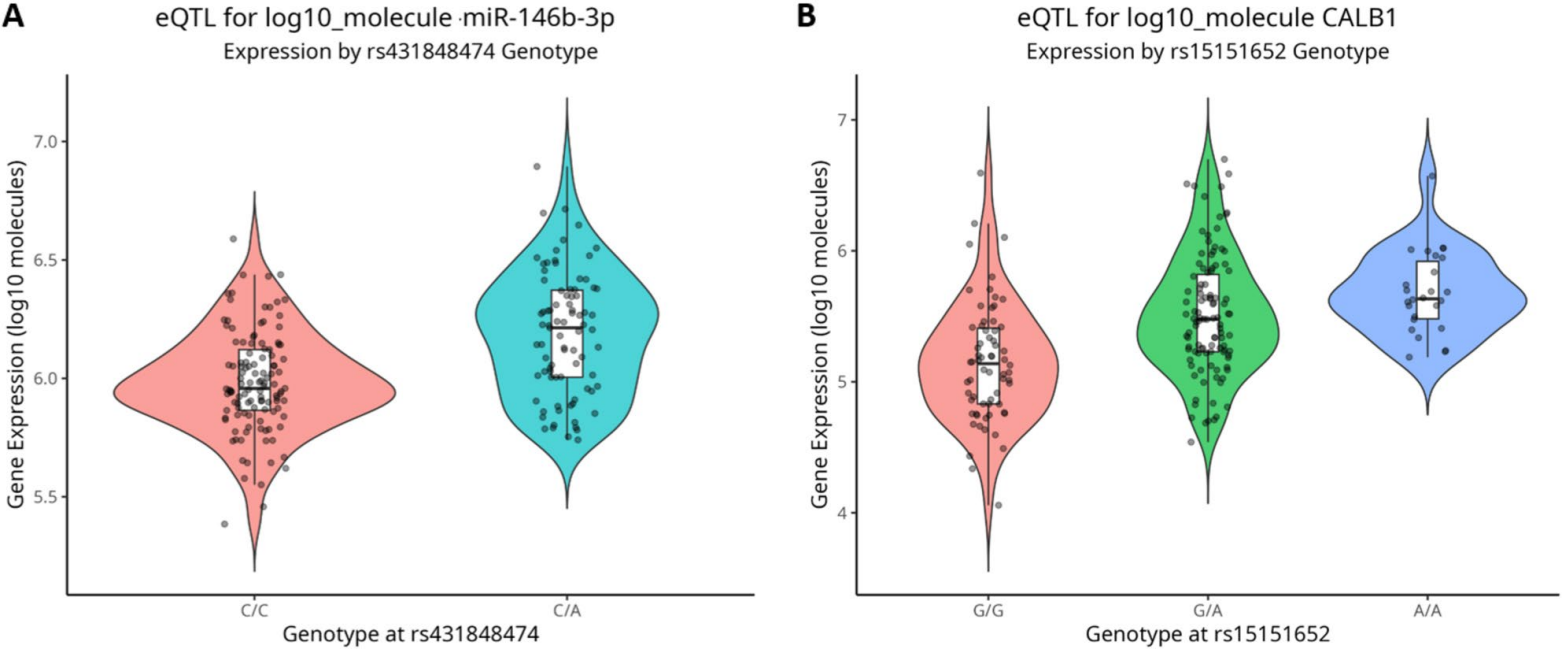
Association of variant-gene expression. Violin plot showing (**A**) miR-146b expression of rs431848474 in Lohmann Selected Leghorn (LSL) hens, (**B**) *CALB1* expression by rs15151652 genotypes in the jejunum mucosa in Lohmann Brown (LB) hens

**Fig. 3:**
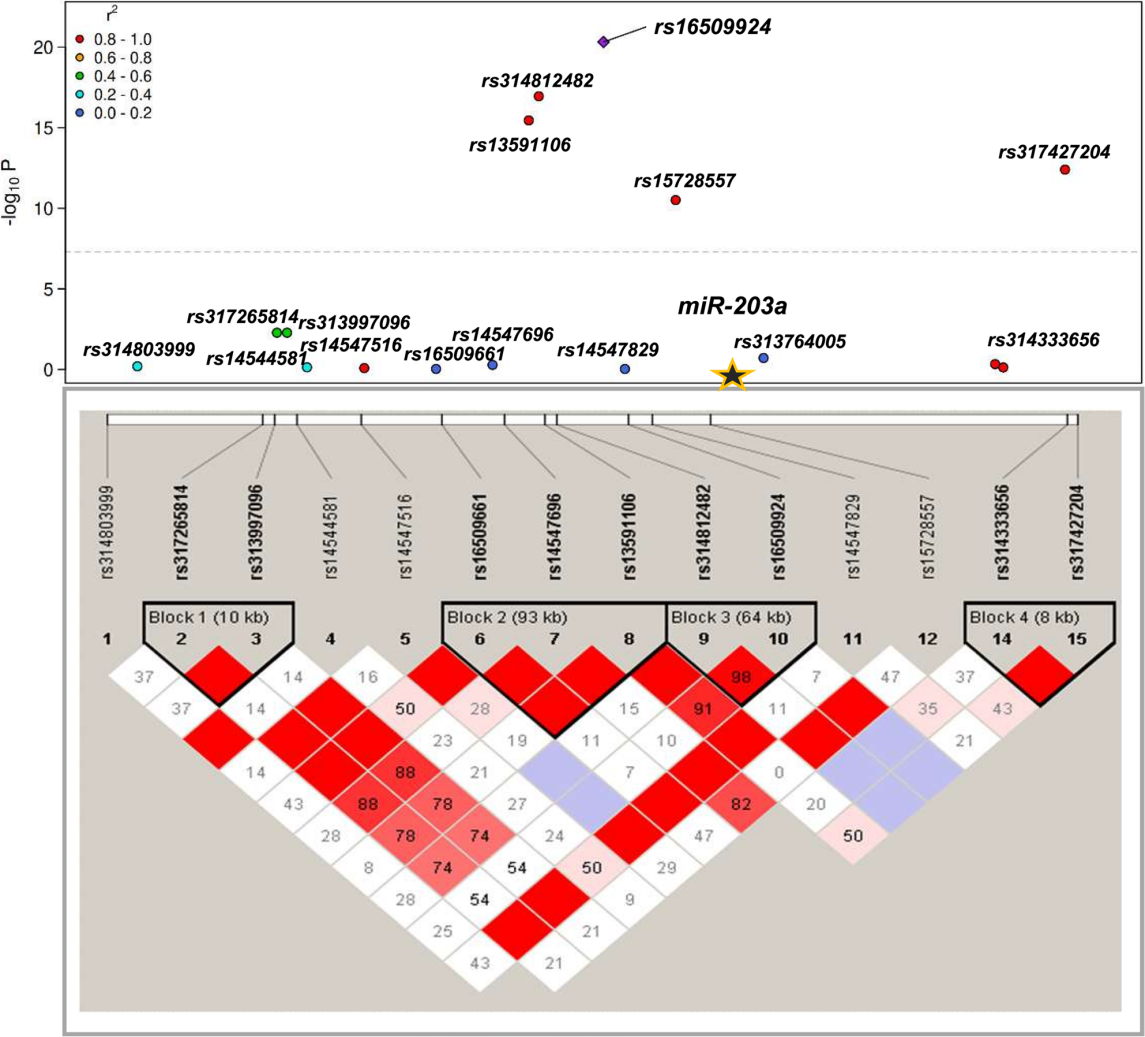
Regional association plot for the gga-miR-203a locus on chromosome 5 identified in the LSL-specific analysis. The upper panel shows the statistical significance (corresponding to -log₁₀ *P* = 8) of the association for single nucleotide polymorphisms (SNPs) within the genomic region around the gga-miR-203a miRNA transcript. Each dot represents a single SNP. The leading SNP, rs16509924, shows the strongest association signal and is shown in purple. The color of other neighboring SNPs corresponds to their linkage disequilibrium (r²) with rs16509924. The bottom panel shows the haplotype block structure and pattern of linkage disequilibrium of variants surrounding the miR-203a region

The LSL strain exhibited a strong *cis*-eQTL cluster on Chr 5 for miR-203a, with 23 total *cis* hits, including rs16509924, rs314812482, and rs13591106, and on Chr 6 for miR 146b-3p [Additional file 1 - Table S2]. The results revealed a clear genetic influence on the expression of miR-146b, with several *cis*-SNPs regulating miR-146b. For example, the [A] allele of the SNP rs431848474 was associated with significantly higher jejunal expression in LSL hens with effect size β = +0.30 (Fig. 2A). The most significant associations in LSL were located at Chr 6 (32 of 58), reflecting the strong effect of markers in *cis* on jejunal miR-146b expression (Fig. 1B). The top *cis* peak was miR-203a at SNP rs16509924 (Chr 5: 50,756,349 bp; p_wald = 4.85 × 10⁻²¹, β = -0.276, FDR = 4.99 × 10^− 17^). In LSL hens, the minor allele of the SNP rs16509924 was associated with significantly lower expression (β = -0.27). On the other hand, the SNP rs15728557 was associated with significantly increased expression of the target transcript with the effect allele [G] (β = +0.18). This locus corresponds to a small region with many SNPs in high linkage disequilibrium with the lead SNP (Fig. 3). In total, 35 SNPs exhibited significant *trans* associations, influencing four miRNAs (Table 1). *Trans*-acting eQTLs were enriched on Chr 1 (miR-1788-5p), Chr 6 (miR-146b-3p), Chr 19 and 23 (miR-30a-5p).

The combined-strain results revealed a total of 104 miR-eQTLs, which together regulated the expression of eight different miRNAs (Table 1). In total, this includes 21 *cis*-miR-eQTLs affecting two specific miRNAs, namely, miR-7460 and miR-146b [Additional file 1 - Table S2]. The most significant association with a SNP marker in *cis* was found for miR-7460 at SNP rs14058862 (Chr 13: 1,0586,210 bp; p_wald = 1.91 × 10^− 15^, β = +0.420, FDR = 3.9 × 10^− 11^) (Fig. 1C).

### eQTLs for mRNAs (mRNA-eQTL)

**Fig. 4:**
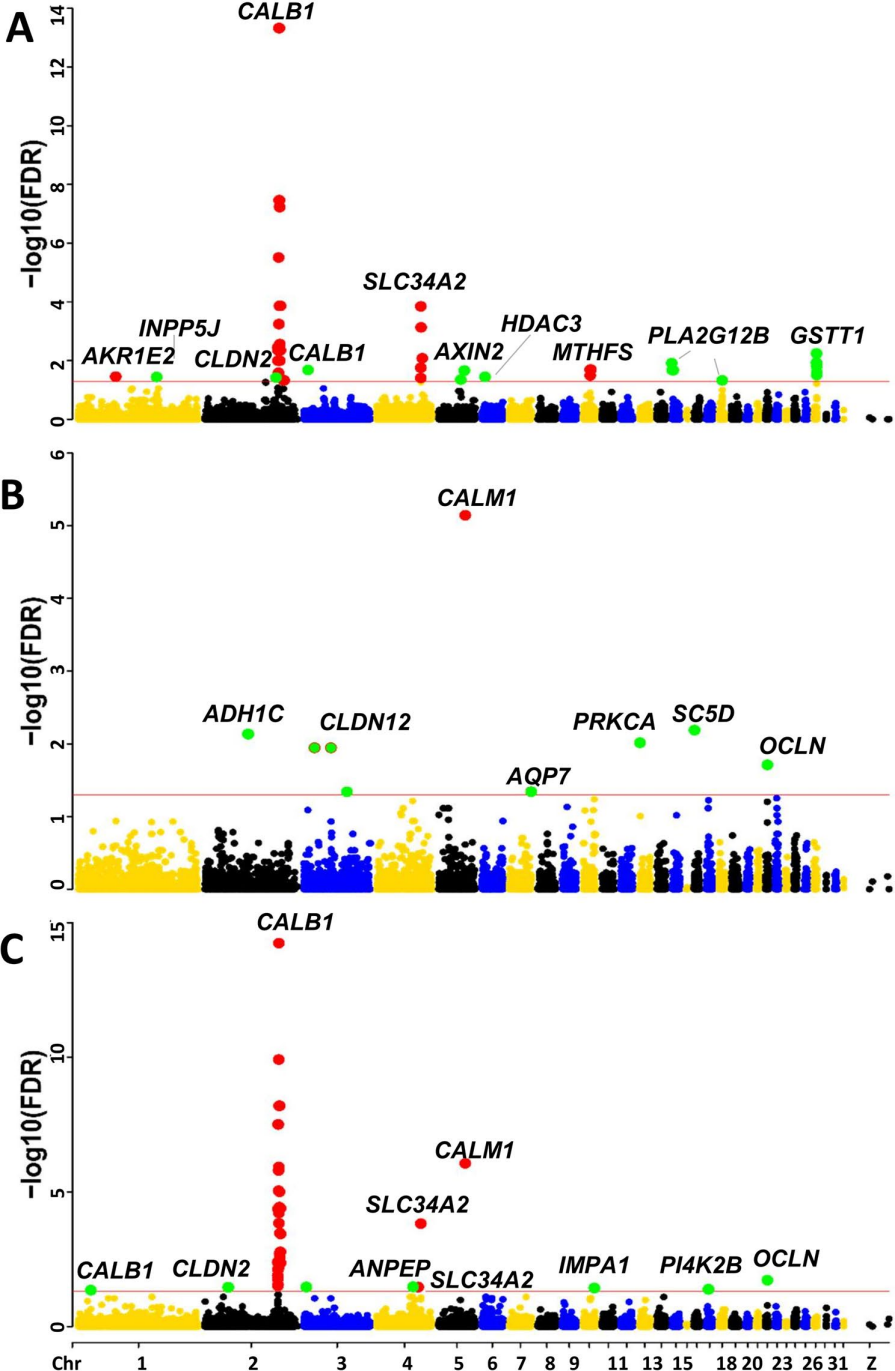
Manhattan plot of mRNA quantitative trait loci (mRNA-eQTLs) for 65 highly expressed jejunal transcripts laying hens. Panels display results for the (**A**) Lohmann Brown (LB) strain (*n* = 199), (**B**) Lohmann Selected Leghorn (LSL) strain (*n* = 200), and (**C**) a combined analysis for both strains with the strain included as a covariate. Red points indicate significant *cis*-eQTLs (variants within 1 Mb of the target gene), whereas green points denote significant *trans*-eQTLs. The horizontal red line represents the genome-wide significance threshold (false discovery rate (FDR) < 0.05), whereas the x-axis represents the chromosomal position according to the GRCg6a assembly

Using the same linear mixed model framework (GEMMA) as for miRNAs, we tested expression phenotypes against 37,611 genome-wide SNPs separately for both LB and LSL hens. At an FDR threshold of < 0.05, we detected 94 and 29 significant mRNA‒SNP associations in LB and LSL, respectively (Fig. 4A-B). Table 2 summarizes the number of significant *cis*- and *trans*-mRNA-QTLs identified in each strain, along with the combined strain analysis [see Additional file 1 - Table S3].

**Table 2:** Distribution of significant mRNA-eQTLs by strain and regulatory type

Strain	Total	cis	Unique_cis_mRNAs	trans	Unique_trans_mRNAs
LB	94	24	*AKR1E2*, *CALB1*, *MTHFS*, *SLC34A2*	70	*AXIN2*, *CALB1*, *CLDN12*, *GSTT1*, *HDAC3*, *PLA2G12B*, *SLC34A2*
LSL	29	2	*CALM1*	27	*ADH1C*, *CLDN12*, *OCLN*, *PRKCA*, *SC5D*
Both	52	16	*CALB1*,* SLC34A2*	36	*CALB1*,* CLDN2*,* IMPA1*,* OCLN*,* PI4K2B*,* ANPEP*

The columns indicate the hen strain, the total number of significant associations, the number of *cis*-and*trans*-acting eQTLs, and the corresponding distinct transcripts targeted by these associations. The genome-wide significance threshold was set to false discovery rate (FDR) < 0.05

^a^*LB *Lohmann Brown, *LSL* Lohmann Selected Leghorn hen strains, and the combined strain analyses (Both)

**Fig. 5:**
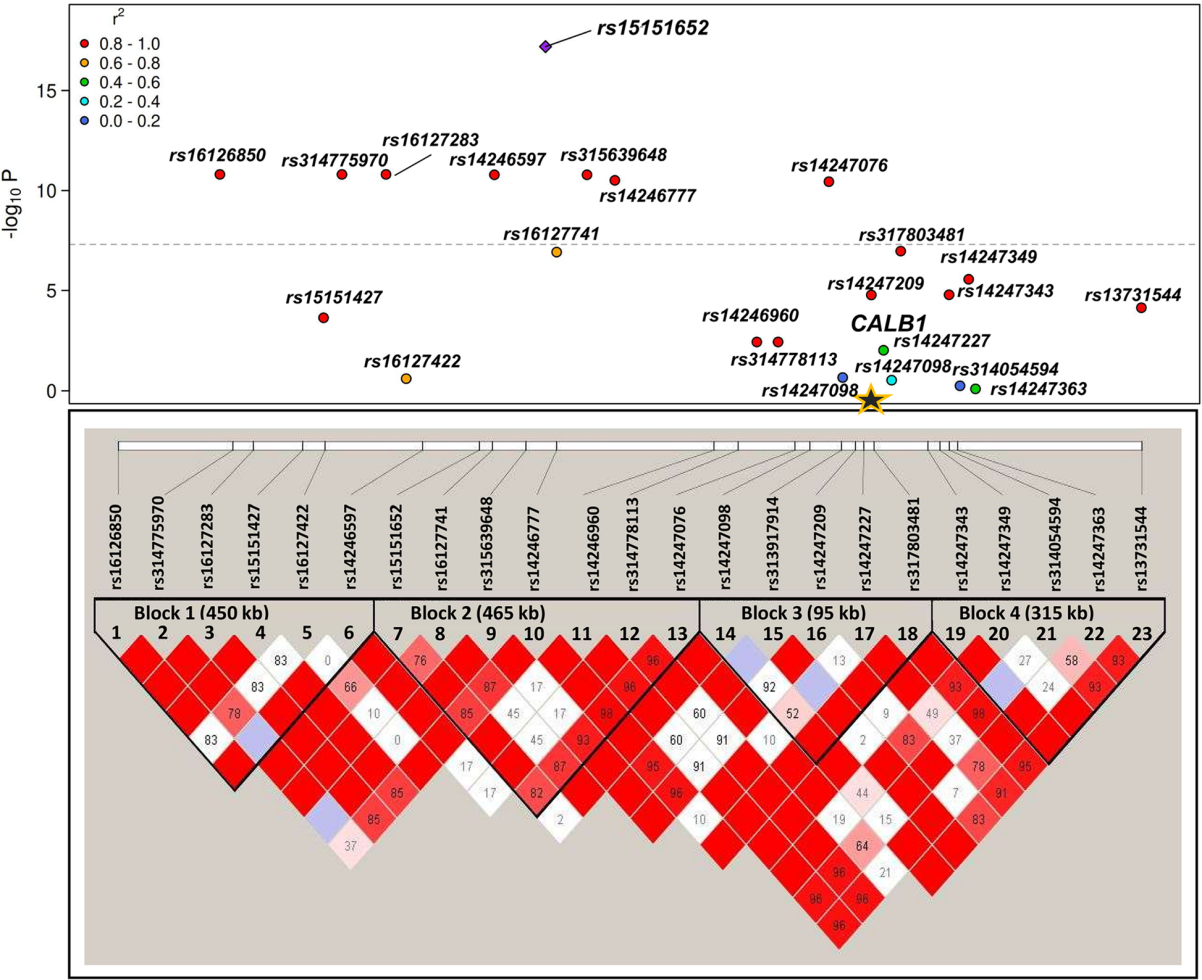
Regional association plot for the *CALB1* locus on chromosome 2 identified in the LB-specific analysis. The upper panel shows the statistical significance (corresponding to -log₁₀ *P* = 8) of the association for single nucleotide polymorphisms (SNPs) within the genomic region around the *CALB1* gene. Each dot represents a single SNP. The leading SNP, rs15151652, shows the strongest association signal and is shown in purple. The color of other neighboring SNPs corresponds to their linkage disequilibrium (r²) with rs15151652. The bottom panel shows the haplotype block structure and pattern of linkage disequilibrium of variants surrounding the *CALB1* region

In LB, a total of 24 SNPs reached genome-wide significance, corresponding to the jejunal expression of four unique genes: *CALB1*, *SLC34A2*, *MTHFS*, and *AKR1E2* (Fig. 4A). Chromosomes 2 and 4 host the majority of the *cis* signals. The strongest *cis* association was observed for *CALB1* at SNP rs15151652 (Chr 2: 123,826,191 bp; p_wald = 6.4 × 10^− 18^, β = +0.323, FDR = 4.70 × 10^− 14^). The effect allele of the SNP rs15151652, which is the lead *cis*-regulatory SNP for *CALB1* expression in LB hens, was associated with significantly higher expression than the alternative allele with effect size β = +0.32 (Fig. 2B). Therefore, carriers of the effect allele were associated with higher CALB1 expression, which may indicate a potential role in intestinal calcium handling. We identified 28 significant *trans*-acting SNPs associated with the expression of seven putative transcripts (including *CALB1*, *AXIN2*, *CLDN12*, *GSTT1*, *HDAC3*, *PLA2G12B*, and *SLC34A2*). *CALB1* and *SLC34A2* are under strong local (*cis*) regulation, with multiple SNPs within 1 Mb of the gene’s transcription start site, while the same genes also show distal (*trans*) associations with other loci (Table 2) [see Additional file 1 - Table S3]. The SNP variants associated with the *CALB1* phenotype were in high linkage disequilibrium with the lead SNP rs15151652. Seven significant SNPs presented a r² value greater than 0.8 (Fig. 5).

In LSL, almost all significant eQTLs were distal regulators, with only *CALM1* showing local regulation on Chr 5 with two associated SNPs (rs14540324 and rs14540338). The strongest *cis*-association was revealed for *CALM1* at SNP rs14540324 (Chr 5: 44,067,483 bp; p_wald = 1.39 × 10^− 09^, β = -0.206, FDR = 7.15 × 10^− 06^). Conversely, 23 significant *trans*-acting SNPs associated with the expression of five distinct transcripts, including *ADH1C*, *CLDN12*, *OCLN*, *PRKCA*, and *SC5D*, were identified (Fig. 4B).

The combined-strain results revealed 52 eQTLs, which together regulated the expression of eight different mRNAs (Table 2) [see Additional file 1 - Table S3]. In total, this comprised 16 *cis*-eQTLs linked to three of these transcripts, namely, *CALB1*, *CALM1*, and *SLC34A2*. The most significant association with a SNP marker in *cis* was observed for *CALB1* at SNP rs16127283 (Chr 2: 123,491,968 bp; p_wald = 2.68 × 10^− 12^, β = +0.355, FDR = 6.46 × 10^− 09^). The results of the strain-specific and combined strain analyses largely overlapped (Fig. 4C).

### Microbiota composition

The 10 miRNAs showing the strongest correlation with the CLR-transformed microbiota data were identified. These represented the top-ranked transcripts based on the strength of association and biological relevance in the respective analyses. These top 10 miRNAs included miR-7460-3p, miR-7460-5p, miR-1b-3p, miR-23a-3p, miR-24-3p, miR-23b-3p, miR-146c-3p, miR-726-5p, miR-16-5p, and miR-133b [see Additional file 1 - Table S4]. All these miRNAs, particularly miR-7460-3p and miR-7460-5p, were significantly positively correlated with *Loigolactobacillus backii* and *Lactobacillus* species, including *L. acidophilus*,* L. amylovorus*, and *L. ultunensis.* In contrast, they exhibited significant negative correlations with *Streptococcus* species such as *S. pluranimalium*,* S. equinus*, and *S. agalactiae*, as well as with *Turicibacter* species (*TJ11* and *T. bilis*) and *Escherichia coli*.

The top 10 mRNAs and microbial species showing the strongest correlations were identified in the same way, based on association strength and biological relevance in the respective analyses. The selected mRNAs included *ADH1L*, *ADH6*, *ST14*, *ADH1C*, *ETNPPL*, *IMPAD1*, *PIP4K2B*, *PI4K2B*, *GPT2*, and *SC5D* [see Additional file 1 - Table S5]. Significant positive correlations were detected between the expression of *ADH1L*, *ADH6*, *ADH1C*, *ETNPPL*, *PIP4K2B*, *PI4K2B*, *GPT2*, and *SC5D* and the abundance of *Streptococcus alactolyticus*, as well as *Turicibacter* species, including *TJ11*,* T. bilis*, and *T. H121*. In contrast, jejunal expression of *ST14* and *IMPAD1* exhibited strong negative correlations with the same *Turicibacter* species (*TJ11*,* T. bilis*, and *T. H121*) and with *S. alactolyticus* and *S. equinus*.

**Fig. 6:**
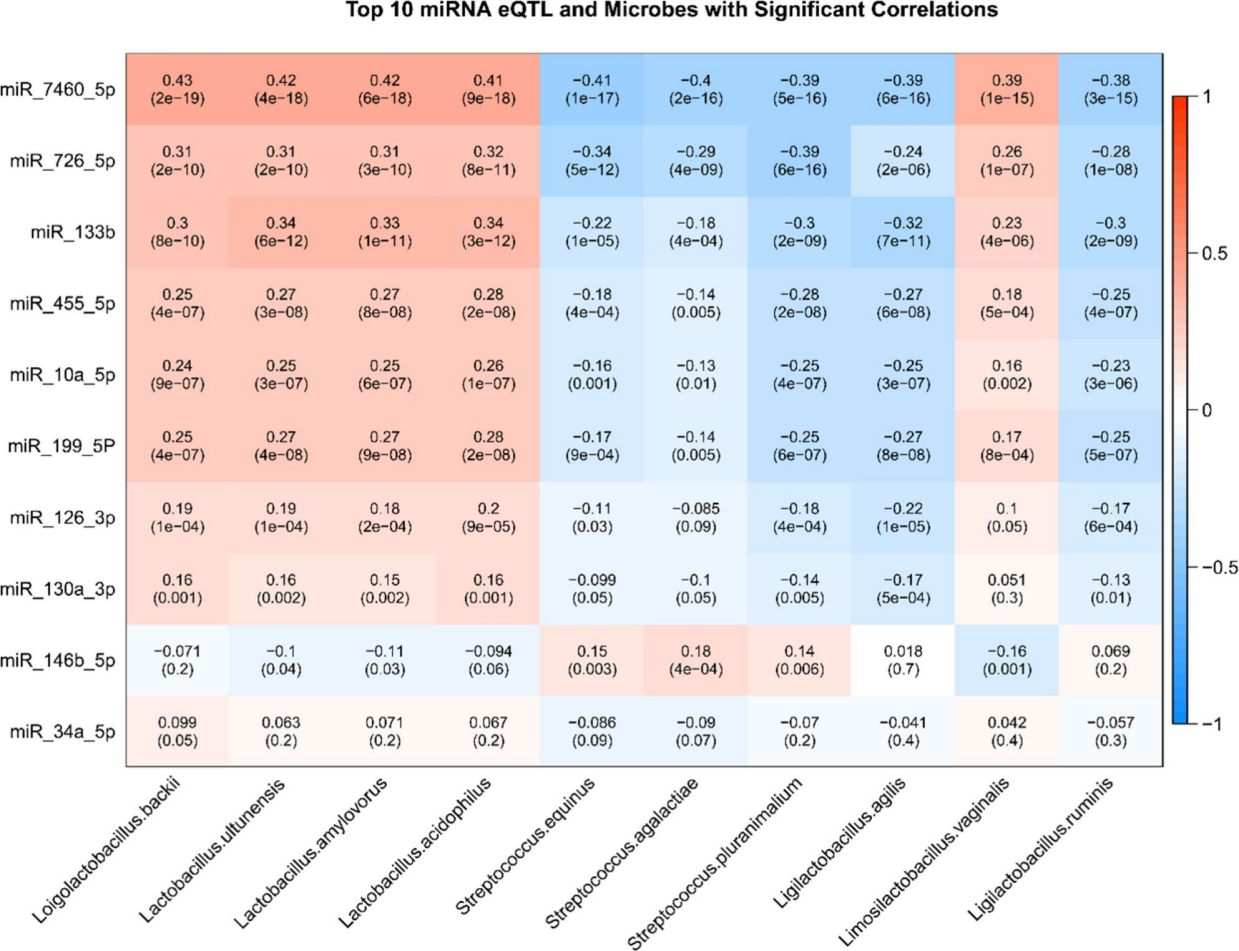
Heatmap of Spearman correlation coefficients and *P* values (in parentheses) between residuals of microbiota and miRNA expression. Correlations were calculated between microbial species abundance and the expression of the ten miRNAs with the most significant *cis*-eQTLs (y-axis). Analysis was performed on data from both strains. The color of each cell denotes the direction and magnitude of the correlation (dark red = strong positive correlation, dark blue = strong negative correlation)

**Fig. 7:**
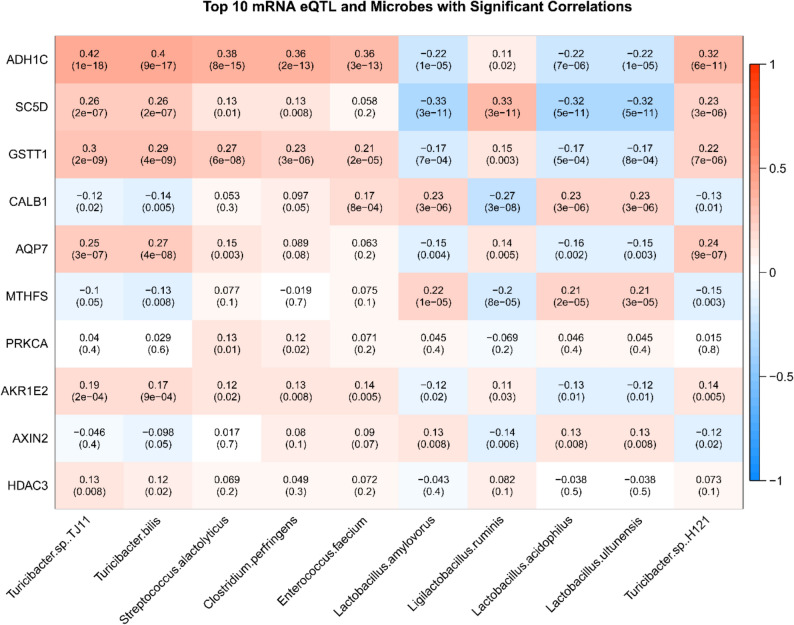
Heatmap of Spearman correlation coefficients and *P*s (in parentheses) between microbiota and mRNA expression. Correlations were calculated between microbial species abundance and the expression of the ten mRNAs with the most significant *cis*-eQTLs (y-axis). Analysis was performed on data from both strains. The color of each cell denotes the direction and magnitude of the correlation (dark red = strong positive correlation, dark blue = strong negative correlation)

Furthermore, genetic regulation of miRNAs (miRNA-eQTLs), including miR-7460-5p, miR-726-5p, miR-133b, miR-455-5p, miR-10a-5p, miR-199-5p, miR-126-3p, miR-130a-3p, miR-146b-5p, and miR-34a-5p, was evaluated per strain and in the combined dataset [see Additional file 1 - Table S6]. Most of them were highly significantly correlated with *Loigolactobacillus backii*,* Limosilactobacillus vaginalis* and *Lactobacillus* species *L. ultunensis*,* L. amylovorus*, and *L. acidophilus*, while a significant negative correlation was found with *S. equinus*,* S. agalactiae*, and *S. pluranimalium and Ligilactobacillus* species *L. agilis* and *L. ruminis*, except for miR-146b-5p and miR-34a-5p (Fig. 6). The top 10 mRNA-associated eQTLs, identified both within each strain and in the combined dataset, included *ADH1C*, *SC5D*, *GSTT1*, *CALB1*, *AQP7*, *MTHFS*, *PRKCA*, *AKR1E2*, *AXIN2*, and *HDAC3* [see Additional file 1 - Table S7]. Among these, *ADH1C*, *SC5D*, and *GSTT1* presented strong positive correlations with *Turicibacter* species *T. TJ11* and *T. bilis*, *Streptococcus alactolyticus*,* Clostridium perfringens*,* Enterococcus faecium*,* Ligilactobacillus ruminis*, and *Turicibacter* sp. *H121*. *CALB1* was significantly positively correlated with *Lactobacillus* species, including *L. amylovorus*,* L. acidophilus*, and *L. ultunensis*. Similarly, *AQP7* exhibited a significant positive correlation with *Turicibacter* species (Fig. 7).

## Discussion

This study was built upon the findings of previous research, which showed that low-P diets trigger marked transcriptional shifts in the small intestine of LB and LSL hens [10, 24]. Because expression-related QTLs provide a locus-specific link between DNA variation and transcription [36], we used an integrated approach to first map *cis*- and *trans*-eQTLs for the same low-P responsive miRNAs and mRNAs in these two laying hen strains and, second, to test for correlations between these genetically regulated transcripts and the gut microbiota. With this approach, we aimed to determine how segregating sequence polymorphisms influence the host’s gut transcriptome and to explore the relationships between polymorphisms, the host’s gut transcriptome, and its microbiome composition. Large multi-tissue chicken eQTL atlases reveal that hundreds of intestinal transcripts carry significant *cis*-eQTLs under standard diets (ChickenGTEx Consortium, 2023) [37].

The eQTL analysis identified local (*cis*) and distal (*trans*) regulatory variants. However, *cis*-eQTLs were more numerous and typically exhibited larger effect sizes on gene expression than the detected *trans*-eQTLs. This finding is consistent with established results in genetical genomics [38–40]. *Cis*-eQTLs represent a direct link between a genetic variant and the expression of a target gene, resulting in consistent and direct effects on transcription [41]. This allele variation within important regulatory elements, such as promoters or enhancers, provides a strong and localized signal that can be more easily detected with a high statistical significance threshold. In contrast, *trans*-acting variants have an indirect influence on gene expression, typically through intermediary molecules, such as transcription factors and/or miRNAs, which may lead to diffuse, smaller effects distributed across many regions of the genome [42].

Among the most significant *cis*-miRNAs, two candidate quantitative trait miRNAs, namely, miR-146b and miR-203a, were found to be conserved in both strains. MiR-146b and miR-203a appear to be relevant to phosphorus-related physiology mainly through their roles in kidney-gut-bone mineral metabolism rather than through direct, canonical regulation of phosphate transport. For miR-146b, there are links to bone and kidney pathology relevant to phosphate balance; for miR-203a, the main evidence points to osteogenic differentiation and bone-related signaling rather than a direct phosphate-transport role [43–45].

Carriers of the rs431848474 SNP might be associated with a more robust immunomodulatory response, given the known function of miR-146b, which has been shown to target the *AKT1* gene in chickens and thus regulate apoptosis in e.g., chicken granulosa cells [46]. For example, G. Hu et al., (2022) reported that *Salmonella enterica* infection activates miR-146b-5p expression in chicken cecum tissues [47]. Ponsuksili, Oster, et al., (2021) also identified miR-146b-5p as one of the top transcripts associated with intestinal P utilization and immune pathways in an experimental quail population [19]. Specifically, in chicken myoblasts, miR-146b has been experimentally shown to directly target and suppress the expression of *MDFIC* and inhibit the *PI3K/AKT* pathway [48]. Moreover, it is a known role of miR-146b in improving the gut barrier and modulating inflammatory responses [49, 50]. Its upregulation may therefore serve to limit tissue damage and restore homeostasis under dysbiotic conditions.

A strong association for miR-203a was also observed. miR-203a is known to regulate metabolic, immune, and tumour-suppressive processes in the gut, and its expression can be influenced by gut microbiota composition in various clinical conditions [51–53]. In obese rodents, it was shown to reduce obesity and dyslipidemia. The underlying mechanism involves the inhibition of *ASBT*, a direct target of the miRNA, which was demonstrated to decrease bile acid uptake in human intestinal epithelial cells [52]. Microbiota profiling studies have linked *Turicibacter* to host fat metabolism, including adiposity and lipid intake [54, 55]. Recent evidence identifies genes in *Turicibacter* strains that allow them to modify host bile acid composition and lipid metabolism, supporting their role as modulators of host fat biology [56]. In our study, we observed a significant positive correlation between miR-203a and *Turicibacter sp. TJ11* specifically within the LB strain (*r* = 0.27, *P* = 0.0001).

*Cis*-miR-QTLs for miR-146b and miR-203a showed strong signals in both LB and LSL strains, indicating shared regulatory mechanisms. In contrast, distinct *cis*-miR-eQTLs for miR-219b, miR-34a, and miR-7460 highlighted notable strain-specific regulation. This finding is particularly relevant for miR-7460, as the analysis revealed a positive correlation between its expression and *Lactobacillus* species and a negative correlation with *Streptococcus* species. *Lactobacillus* species, notably *L. acidophilus*,* L. amylovorus*, and, to a lesser extent, *L. ultunensis*, are widely recognized for their competitive exclusion of pathogens, ability to maintain gut health, and ability to increase productivity [57]. These bacteria promote the production of short-chain fatty acids (SCFA), and higher SCFA levels in turn support the repair of the intestinal mucosa, energy supply to gut cells, and overall improvements in tight junction integrity [58]. On the other hand, several species within the *Streptococcus* genus, such as *S. pluranimalium*,* S. equinus*, and *S. agalactiae*, are associated with infections in poultry [59]. In this context, the negative correlation with miRNAs may be consistent with a host protective mechanism. A study on chicken caecal responses to *Histomonas meleagridis* infection implicates gga‑miR‑7460‑3p in activating Hedgehog signaling via the transcription factor *Gli2*, suggesting a possible role in gut mucosal or immune processes [60].

The strain-specific regulation of miRNA expression identified in this study may offer a molecular explanation for previously reported physiological differences between LB and LSL hens, including variations in their metabolic pathways, immune systems, and microbiota composition [10, 61, 62]. These regulatory differences were apparent at both the local (*cis*) and distal (*trans*) levels. For local effects, *cis*-eQTLs were found for miR-146b and miR-203a in both strains, while others were exclusively identified in LB hens, such as those regulating miR-219b, miR-34a, and miR-7460. In addition to these local effects, the regulatory mechanism in each strain appears to be actively regulated through distal mechanisms, with the presence of unique sets of *trans*-acting miR-QTLs in the strain-specific analysis, suggesting distinct regulatory pathways. For example, miR-10a, miR130a, and miR-133b were significantly associated only in LB, whereas miR-146b, miR-30a, and miR-1788 were significantly associated exclusively in the LSL. The combined-strain analysis also revealed overlapping results with the strain-specific result. The regulatory function of miR-133b may also be linked to influence the intestinal ecosystem, as its expression was positively correlated with health-promoting *Lactobacillus* species. We identified miR-146b as a highly significant and conserved *cis*-eQTL in both strains. Interestingly, we also found *trans*-acting variants for miR-146b unique to the LSL strain, suggesting an additional layer of distal regulation that may contribute to the distinct immune modulation phenotype of LSL hens. It is well known that strain adaptation to low P feeding is different. While LB hens show greater metabolic adaptation, LSL hens exhibit more pronounced immune modulation [63]. The *trans*-regulatory landscape also reflects this phenomenon. For example, miR-10a has been identified among differentially expressed genes between pullets and mature laying hens and is suggested to be involved in the metabolic shift that occurs with the onset of egg laying in chicken hepatic lipid metabolism [64].

In this study, LB hens showed a higher number of local-acting regulatory variants (*cis*-eQTLs) affecting genes related to mineral metabolism. Four *cis*-eQTLs were identified for *SLC34A2*, *CALB1*, *AKR1E2*, and *MTHFS*. Among the gut-expressed genes, *SLC34A2* showed the clearest link to phosphorus metabolism because of its established role in intestinal phosphate absorption [65–67], whereas *CALB1* may contribute more indirectly through mineral transport and buffering [68]. In contrast, *AKR1E2* and *MTHFS* did not show a clear direct annotation for phosphate metabolism. However, because *MTHFS* is involved in one-carbon metabolism, it may still contribute indirectly to mineral homeostasis through broader metabolic or epigenetic mechanisms that could influence bone matrix synthesis [69]. Together, these *cis*-eQTLs highlight genetic control over key components of intestinal P and calcium handling. Other eQTL-associated transcripts, such as *ADH1C*, *SC5D*, *GSTT1*, and *AKR1E2*, do not currently have established direct roles in P metabolism. However, their involvement in gut mucosal homeostasis, detoxification, and lipid metabolism may indirectly support the intestinal environment required efficient mineral handling. These findings suggest a broader network of genetically regulated processes that may collectively contribute to P utilization efficiency, warranting further functional investigation. In line with this study, LB hens presented a significantly greater expression rate of *SLC34A2* in the ileum than LSL hens fed low-P and low-calcium diets [8]. It appears that the LSL genetic foundation was associated with *CALM1* expression, suggesting that the two commercial lines differ not only in genetic background and previously reported traits, such as bone quality and mineralization [70], mineral utilization mechanisms [63], and gene expression profiles [10, 11, 24] but also in the local regulatory variation that drives mineral utilization phenotypes. Notably, the majority of the eQTLs presented strain-specific patterns. This finding suggests the possibility of distinct architectures for jejunal-specific gene expression in LB and LSL. In addition to direct mineral transporters, our analysis revealed associations between host metabolic gene expression and the microbiome. For example, a group of host metabolic genes, such as *ADH1C*, *SC5D*, and *GSTT1*, was strongly correlated with the abundances of *Turicibacter* and *S. alactolyticus*. *Turicibacter* is central to bile and lipid metabolism, modulating how the host digests and metabolizes fat via modification of bile acids [56]. Lactic acid bacteria like *S. alactolyticus* enhance SCFA production and support gut epithelial health, which can help mitigate inflammation [71]. *ADH1C*, an alcohol dehydrogenase, likely metabolizes endogenous ethanol from gut microbial fermentation in the avian jejunum, supporting mucosal detoxification and homeostasis. While *S. alactolyticus* relates to protein and SCFA metabolism, and *ADH1C* to alcohol metabolism and inflammation regulation, both support gut barrier integrity and metabolic balance, suggesting a potential link between host genetic variation in these metabolic genes and the abundance of key microbes such as *Turicibacter* and *S. alactolyticus*, although the directionality of this relationship remains to be established. In addition, *S. alactolyticus* is pivotal for protein and amino acid metabolism, especially when dietary protein is limited, and may support host nutrition and gut health via fermentation and enzyme secretion [72]. Both contribute meaningfully, but in different ways, to the overall metabolic functions of the gut ecosystem. It should be noted that the host-microbiome associations reported here are based on Spearman’s rank correlations and do not establish causality. While previous studies have provided evidence for directional relationships between gut microbiota and P efficiency traits in poultry [20, 21], experimental approaches such as mediation analysis would be needed to determine whether genetically regulated host transcripts causally shape microbial composition or vice versa.

## Conclusions

In conclusion, this study provides evidence for distinct differences in the gastrointestinal tracts of LB and LSL hen strains due to specific heritable differences in their gene regulation. Key *cis*- and *trans*-eQTLs were identified that likely govern the strain-specific expression of important metabolic- and immune-related transcripts within the context of a transient low-P diet in mature laying hens. Moreover, the findings showed that the influence of this host genetic architecture extends beyond endogenous pathways. By linking eQTL-regulated genes, such as miR-146b and *CALB1*, to the abundance of key bacterial genera, such as *Lactobacillus*, we propose a functional holobiont in laying hens.

## Supplementary Information

Supplementary Material 1.

## Data Availability

The datasets supporting the conclusions of this article are included within the article and its additional file. The SNPs and phenotype data showing significant associations, including genetic polymorphisms and linked genotype–phenotype information, are available in the electronic supplementary material (Additional file 1, Tables S1–S7). The complete genotype dataset cannot be made publicly available due to restrictions imposed by the consortium data-use agreement governing the use of these data by our project partner.

## Abbreviations

ASV: Amplicon Sequence Variant
CLR: Centered Log-Ratio
Chr: Chromosome
Ct / Cq: Cycle threshold / Quantification cycle
eQTL: expression Quantitative Trait Locus
FDR: False discovery rate
GEMMA: Genome-wide Efficient Mixed Model Association
GRCg6a: Gallus gallus reference consortium genome build 6a
InsP: Inositol Phosphate
LB: Lohmann Brown
LMM: Linear Mixed Model
LSL: Lohmann Selected Leghorn
MAF: Minor Allele Frequency
miR-eQTL: microRNA Expression Quantitative Trait Locus
mRNA-eQTL: messenger-RNA Expression Quantitative Trait Locus
P: Phosphorus
qPCR: quantitative Polymerase Chain Reaction
REML: Restricted Maximum Likelihood
SCFA: Short chain fatty acid
SNP: Single Nucleotide Polymorphism
STA: Specific Target Amplification
